# The spontaneous expulsion of a large colonic lipoma: a rare case

**DOI:** 10.1016/j.igie.2026.01.008

**Published:** 2026-01-24

**Authors:** Shamira Ghouse, Emanuelle Suratos, Dee-Ann Davies, Rajiv Swamy, Carolina Lopez, Vivek Gupta, Sabina Beg

**Affiliations:** East And North Hertfordshire NHS Teaching Trust, Lister Hospital, Stevenage, United Kingdom

A 42-year-old man with no relevant existing medical history was referred for colonoscopy via the urgent suspected cancer pathway because of new symptoms of intermittent abdominal pain and an altered bowel habit. On colonoscopy, a large polypoidal lesion exceeding 50 mm in diameter, causing intussusception, was found in the distal descending colon. The lesion was subepithelial with ulcerated mucosa (Fig. 1A). Multiple biopsy specimens were taken, but no attempt was made at removal. Histology demonstrated ulceration with mucosal prolapse and no evidence of malignancy.

**Figure 1:**
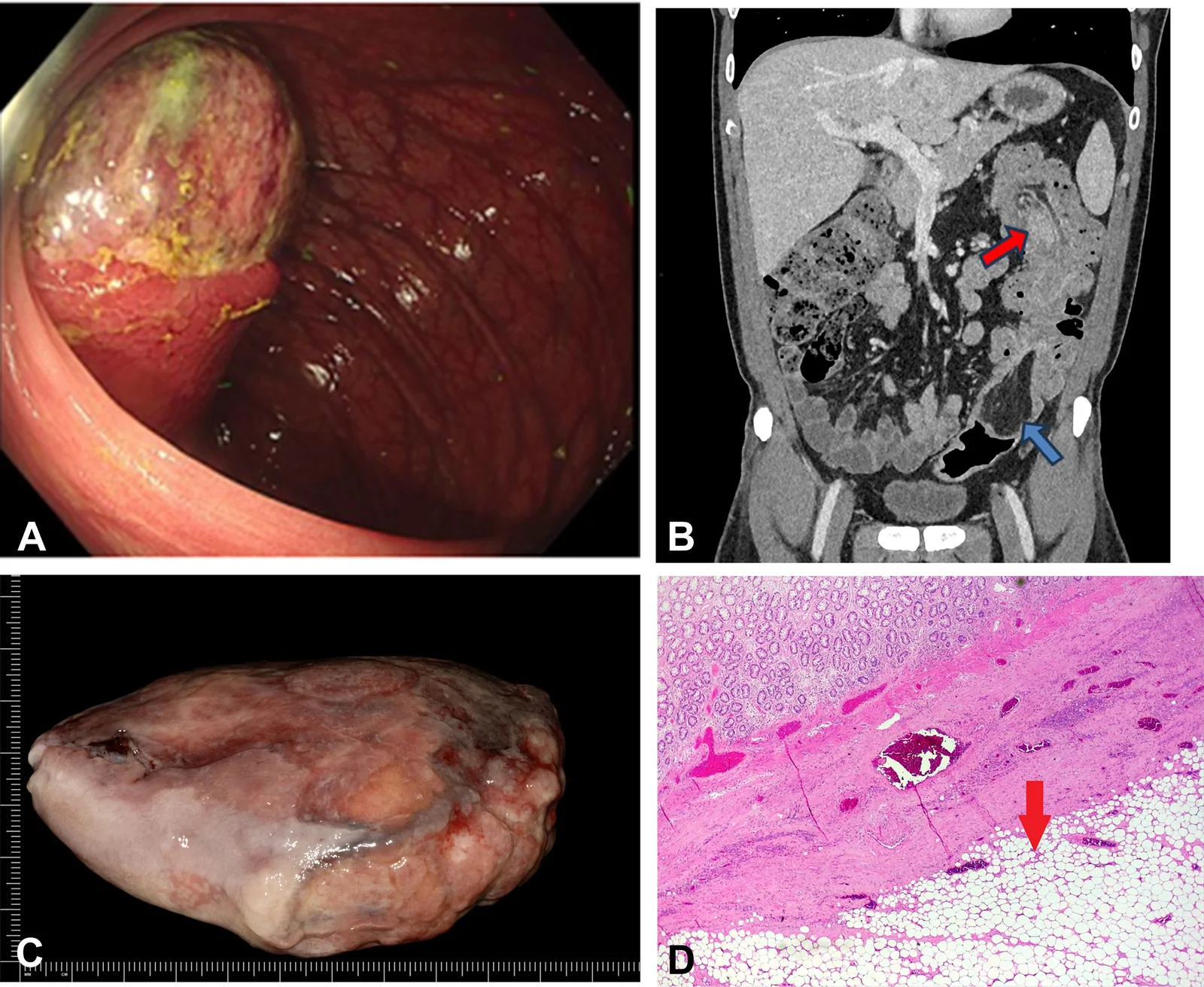
**A,** Endoscopic image from the index colonoscopy demonstrating the subepithelial lesion found. **B,** Coronal cross-sectional imaging showing the mass (*blue arrow*) and the associated segment of intussuscepted bowel (*red arrow*). **C,** The mass that was spontaneously passed by the patient. **D,** Histologic section stained with hematoxylin and eosin, original magnification ×10, showing benign mucosa with submucosal distribution of the lipoma (*red arrow*).

Cross-sectional imaging confirmed a 60-mm polypoidal mass in the distal descending colon with appearances typical of a lipoma and intussusception spanning 13 cm of the bowel (Fig. 1B). A multidisciplinary meeting recommended surgical resection with a left hemicolectomy. However, the patient declined operative intervention and was referred for a trial of endoscopic deroofing of the lipoma.

Forty-eight hours before the planned procedure, and before bowel preparation, the patient reported a short episode of severe pain, followed by passage of a large, pale mass per rectum (Fig. 1C). This occurred 6 weeks after the index colonoscopy. Follow-up colonoscopy revealed an area of breached mucosa with local ulceration at the prior lesion site but no residual lipoma and complete reduction of the intussusception. Histopathologic analysis of the expelled specimen confirmed a submucosal lipoma (Fig. 1D). The patient was reviewed in clinic 4 months later and remained asymptomatic; therefore, no further endoscopic or radiological follow-up was deemed necessary.

Although adult intussusception caused by lipomas is uncommon, spontaneous expulsion of large colonic lipomas, such as in this case, is exceedingly rare, with few reported cases in the literature, to our knowledge. Spontaneous expulsion of a colonic lipoma was first described by Backenstoe et al^1^^,^^2^ in 1940, and since then, only a small number of cases have been reported, almost exclusively as isolated case reports or limited case series. Available reports suggest that expulsion is typically preceded by abdominal pain and bleeding—symptoms that, as in our patient, often resolve after detachment of the lesion. The underlying mechanism remains uncertain; however, the few documented cases indicate a higher likelihood of expulsion in lipomas with long pedunculated stalks, which may predispose to torsion, ischemia, ulceration, and eventual separation. Some cases have been associated with bowel preparation for colonoscopy, although in our patient, the expulsion occurred before any such preparation. Reported management strategies range from conservative observation when expulsion is complete and symptoms resolve to endoscopic or surgical intervention when adverse events arise or diagnostic uncertainty persists.^1^, ^2^, ^3^, ^4^, ^5^

## Patient consent

The patient in this article has given written informed consent to publication of their case details.

## Disclosure

All authors disclosed no financial relationships.
